# Detection of fucosylated extracellular vesicles miR-4732-5p related to diagnosis of early lung adenocarcinoma by the electrochemical biosensor

**DOI:** 10.1038/s41598-024-61060-z

**Published:** 2024-05-16

**Authors:** Shengting Zhu, Jianlin Chen, Lili Yu, Jiawen Li, Shumin You, Yue Zheng, Wanzhen Zhuang, Bin Qiu, Yi Huang

**Affiliations:** 1 Shengli Clinical Medical College, Fujian Medical University, Fuzhou, 350001 China; 2 Department of Blood Transfusion, Fujian Provincial Hospital, Fuzhou, 350001 China; 3 Ministry of Education Key Laboratory for Analytical Science of Food Safety and Biology (Fuzhou University), Fujian Provincial Key Laboratory of Analysis and Detection for Food Safety, Fuzhou, 350108 China; 4 Department of Clinical Laboratory, Fujian Provincial Hospital, Fuzhou, 350001 China; 5 Central Laboratory, Center for Experimental Research in Clinical Medicine, Fujian Provincial Hospital, Fuzhou, 350001 China; 6 Fujian Provincial Key Laboratory of Critical Care Medicine, Fujian Provincial Key Laboratory of Cardiovascular Disease, Fuzhou, 350001 China

**Keywords:** miR-4732-5p, Biomarker, Lung adenocarcinoma, Diagnostic, Electrochemical biosensor, Biochemistry, Cancer, Pathogenesis

## Abstract

Our preliminary investigation has identified the potential of serum fucosylated extracellular vesicles (EVs) miR-4732-5p in the early diagnosis of lung adenocarcinoma (LUAD) by a fucose-captured strategy utilizing lentil lectin (LCA)-magnetic beads and subsequent screening of high throughput sequencing and validation of real-time quantitative polymerase chain reaction (RT-qPCR). Considering the relatively complicated procedure, expensive equipment, and stringent laboratory condition, we have constructed an electrochemical biosensor assay for the detection of miR-4732-5p. miR-4732-5p is extremely low in serum, down to the fM level, so it needs to be detected by highly sensitive electrochemical methods based on the Mg^2+^-dependent DNAzyme splitting nucleic acid lock (NAL) cycle and hybridization chain reaction (HCR) signal amplification. In this study, signal amplification is achieved through the dual amplification reactions using NAL cycle in combination with HCR. In addition, hybridized DNA strands bind to a large number of methylene blue (MB) molecules to enhance signaling. Based on the above strategy, we further enhance our signal amplification strategies to improve detection sensitivity and accuracy. The implementation of this assay proceeded as follows: initially, miR-4732-5p was combined with NAL, and then Mg^2+^-dependent DNAzyme splitted NAL to release auxiliary DNA (S1) strands, which were subsequently captured by the immobilized capture probe DNA (C1) strands on the electrode surface. Following this, abundant quantities of DNA1 (H1) and DNA2 (H2) tandems were generated by HCR, and S1 strands then hybridized with the H1 and H2 tandems through base complementary pairing. Finally, MB was bonded to the H1 and H2 tandems through π–π stacking interaction, leading to the generation of a signal current upon the detection of a potential capable of inducing a redox change of MB by the electrode. Furthermore, we evaluated the performance of our developed electrochemical biosensor assay. The results demonstrated that our assay is a reliable approach, characterized by its high sensitivity (with a detection limit of 2.6 × 10^−17^ M), excellent specificity, good accuracy, reproducibility, and stability. Additionally, it is cost-effective, requires simple operation, and is portable, making it suitable for the detection of serum fucosylated extracellular vesicles miR-4732-5p. Ultimately, this development has the potential to enhance the diagnostic efficiency for patients with early-stage LUAD.

## Introduction

Lung cancer, the most prevalent and deadly malignant neoplasm globally, poses a grave threat to human well-being^1,2^. Lung adenocarcinoma (LUAD) stands the predominant histologic subtype of lung cancer, comprising approximately 40% of all cases^3^. Demonstrating an exceedingly invasive nature, LUAD exhibits remarkably malignant biological behavior, leading to swift metastasis and an unfavorable 5-year survival rate. Hence, early diagnosis is of great significance for LUAD prognosis^4,5^.

The current application of low-dose spiral CT for the early screening of the LUAD patients is hindered by its inability to effectively distinguish them from benign pulmonary nodule < 1 cm, leading to false-positive and over-diagnosis problems^6,7^. Recently, extracellular vesicles cargoes have been demonstrated to be a prospective approach for the LUAD diagnosis. These 30–150 nm membranous extracellular vesicles contain protein, lipid and genetic material, such as DNA, mRNA, and miRNA^8,9^. Among these the miRNAs present in extracellular vesicles show diagnostic potential as they are protected from ribonuclease degradation^10,11^. However, the efficient isolation of extracellular vesicles derived from cancer cells poses a challenge^12^. In our pilot study, we addressed this issue by focusing on the aberrant glycosylation, especially fucosylation modification observed in cancerous tissues and extracellular vesicles of the patients with malignancies^13–15^. Through a fucose-captured strategy using lentil lectin (LCA)-magnetic beads and subsequent screening of high throughput sequencing and validation through reverse transcription quantitative polymerase chain reaction (RT-qPCR), we identified a serum fucosylated extracellular vesicles miRNA, miR-4732-5p, as a promising serological biomarker for the early diagnosis of LUAD^16^.

RT-qPCR is currently applied for the detection of miRNAs in extracellular vesicles^17^. However, this method faces challenges such as expensive equipment, complicated procedure, and stringent laboratory condition. To overcome the limitations of RT-qPCR, a variety of amplification methods, including hybridization chain reaction (HCR)^18^ and catalytic hairpin assembly^19^, in combination with detection technologies like fluorescence^20^ and electrochemistry^21,22^, have been developed to create efficient and simplified approaches for miRNA detection. Among these approaches, electrochemical assays based on affordable and portable characteristics of electrochemical platform have garnered increasing attention as a powerful tool for point-of-care miRNA detection^23,24^. Moreover, the electrochemical biosensors have been reported to possess the advantages of low cost, easy operation, and high sensitivity as well as specificity^25,26^. A nanostructure called nucleic acid lock (NAL) has been developed based on HCR signal amplification, which presents the catalytic properties like DNAzyme. To date, the NALs have been a widely utilized method for signal amplification in the fields of biosensors and molecular diagnostics. It uses the unique structure and complementary sequence of NALs to capture and release the target nucleic acid sequence, thereby improving the sensitivity and reliability of detection. Based on this concept, we have developed a novel electrochemical biosensor that relies on Mg^2+^-dependent DNA enzyme-assisted cleavage of NAL cycling and HCR signal amplification for the detection of miR-4732-5p in serum extracellular vesicles. We have also conducted studies on the electrochemical workstation, aiming to explore a new detection approach for early diagnosis of lung adenocarcinoma and provide novel insights and methods for the detection of glycosylated vesicular miRNA.

## Materials and methods

### Study participants

A total of 36 hospitalized patients from Fujian Provincial Hospital between January 2022 and December 2022 were enrolled as participants in this study, including 18 individuals with LUAD at stage I, and 18 patients with BPN (Table 1). The tumor stage of LUAD was estimated according to TNM classification of the International Association for the Study of Lung Cancer (IASLC) eighth edition^27^. This study was conducted in accordance with the Code of Ethics of the World Medical Association (Declaration of Helsinki) and approved by the institutional review board (IRB) of Fujian Province hospital (ID: K2018-12-040).

**Table 1:** Clinical data of the participants.

Characteristics		LUAD (Stage I)	BPN
Number		18	18
Age		55.06 ± 10.56	57.33 ± 12.51
Gender	Men	4 (22.2%)	14 (77.8%)
	Women	14 (77.8%)	4 (22.2%)

In this study, 5 ml of peripheral blood from each subject was collected and the serum was separated at 800 g for 5 min and stored at − 80 °C until use.

### Extracellular vesicles and EVs-derived RNA isolation

The fucosylated extracellular vesicles separation method is described in our previous report^28^, as detailed in Supplementary Table 1. Isolation of serum extracellular vesicles and EVs-derived RNAs were performed using an automatic instrument (Hotgen Biotech, Beijing, China, Purification system P48). Serum extracellular vesicles extraction: 500 μl LCA-coupled magnetic bead solution (MBL) and 250 μl serum samples were added per well to the 1st and 7th column of 96-well polypropylene deep well plate (Changzhou Genmag Biotechnology Co., LTD.), 600 μl washing solution (WBL) was added per well to the 2nd and 8th column, and 250 μl elution buffer (EBL) was added per well to the 3rd and 9th column. Subsequently, the extraction procedure was run (Supplementary Table 2), the contents of the 6th and 12th columns of the 96-well polypropylene deep well plate were extracted extracellular vesicles. For EVs-derived miRNA isolation, 800uL lysate (LB), 500 μl washing solution 1 (WB1), 800uL washing solution 2 (WB2), 50 μl elution buffer (EB) were added to the 1st, 2nd, 3rd, 6th column and 7th, 8th, 9th, 12th column of the 96-deep well plate, respectively. Then, 200 μl of extracellular vesicles and 16 μl magnetic bead solution (MB) were added into the 1st and 7th column wells. Finally, the contents of the 6th and 12th column of the 96-deep well plate were the EVs-derived nucleic acid. The concentrations of RNA were quantified using a NanoQ Micro-volume Spectrophotometer (Bioptic Inc., LA, USA).

### Transmission electron microscopy

4 μl of exosome suspension was dropped on the copper mesh and left it for 1 min, and the excess liquid from the edge of the mesh was sucked with filter paper. Then, the negative staining solution (0.5% uranium acetate aqueous solution, pH 4.5) was dropped on the center of the mesh for 1 min, and the excess solution from the edge of the mesh was sucked with filter paper. After drying in the air, the serum extracellular vesicles were imaged by transmission electron microscopy (TEM) on the FEI Tecnai Spirit (100 kV).

### Nanoparticle tracking analysis

Extracellular vesicles were analyzed by a Nano sight NS 300 system (Nano Sight Technology, Malvern, UK) with a 488-nm laser and a highly sensitive camera. First, extracellular vesicles isolated from serum were diluted 100- to 1000-fold with PBS solution and manually injected into the sample chamber at ambient temperature. Extracellular vesicles were measured on the camera in triplicate with an acquisition time of 30 s and the detection threshold was set to 7. Finally, nanoparticle tracking data of extracellular vesicles were analyzed using the nanoparticle tracking analysis (NTA) analytical software (version 2.3).

### Western blotting

The protein concentration of the exosome solution was quantified by a bicinchoninic acid (BCA) kit (Pierce) 23227). The protein samples were mixed with loading buffer and then denatured in boiling water for 10 min. Next, the protein samples were separated by electrophoresis (SDS-PAGE; 12.5%) performed on a sodium dodecyl sulfate–polyacrylamide gel, and the protein samples were then transferred onto polyvinylidene fluoride (PVDF) membranes. After blocking with 5% skimmed milk for 1 h, the PVDF membranes were washed three times with saline containing 0.2–0.4% Tween-20 Tris-buffered saline (TBST). Subsequently, the PVDF membranes were incubated with the antibodies as follows: 1:1000 dilution for anti-CD9 (20597-1-AP, Proteintech), 1:2000 dilution for anti-CD63 (ET1607-2, Huabio, Hangzhou, China), 1:2000 dilution for anti-CD81 (ET1611-87, Huabio, Hangzhou, China), and 1:5000 dilution for anti-Calnexin (10427-2-AP, Proteintech) at 4 °C overnight, respectively. After washing three times with TBST, the PVDF membranes were incubated with horseradish peroxidase-conjugated goat anti-rabbit IgG at room temperature for 1 h. Finally, the immunoreactive bands were imaged by enhanced chemiluminescence reaction, and later placed into a fluorescent chemiluminescence imager to take photographs.

### RT-qPCR assays

TIANGEN® miRcute Kit (KR211) was utilized to synthesize the first strand cDNA. The RT-qPCR of synthesized cDNA was performed by miRcute plus miRNA qPCR kit (FP411, Qiagen) and on the ABI 7500 real-time PCR system (Applied Biosystems, Foster City, CA, USA) with the following parameters: pre-denaturation at 94 °C for 2 min, followed by 40 cycles of denaturation at 94 °C for 10 s, annealing at 60 °C for 34 s. The relative expression of miRNA was quantified using the 2^−∆∆Ct^ method and miR-20a was served as the internal reference^28^. Primers were synthesized by Shangya Biotechnology (Fuzhou, China) and presented in Table 2.

**Table 2:** List of primers. (F: upstream primers).

Primer names	Primer sequences (5′–3′)
hsa-miR-4732-5p-F	CTGTAGAGCAGGGAGCAGGAAG
hsa-miR-20a-F	CCGCGTAAAGTGCTTATAGTGCAGGTAG

### Electrode pretreatment and electrochemical biosensor fabrication

All DNA strand sequences and miRNAs (miR-4732-5p, SMT, DMT, TMT, miR-92a-3p and miR-1180-3p) sequences were synthesized by Fuzhou Shangya Biotechnology Co (Table 3). Among them, our previous study demonstrated that the expression of extracellular vesicles-derived miR-92a-3p and miR-1180-3p was significantly higher in LUAD than in BPN patients, and thus was used to analyze the specificity of electrochemical biosensor bioassay.

**Table 3:** List of Sequences.

Names	Sequences
NAL	3′-GACATCTCGTTGCTCTCGGCCTGCTTGCG
	TTTTTGAGCGCATrAAAGCGCTCGTCCT-5′
Capture probe C1	3′-HS-(CH)6-TTTT-AGGACGA-5′
H1	3′-GCGCTTCACATA-5′
H2	3′-GTGTATCGCGAA-5′

**Figure 1:**
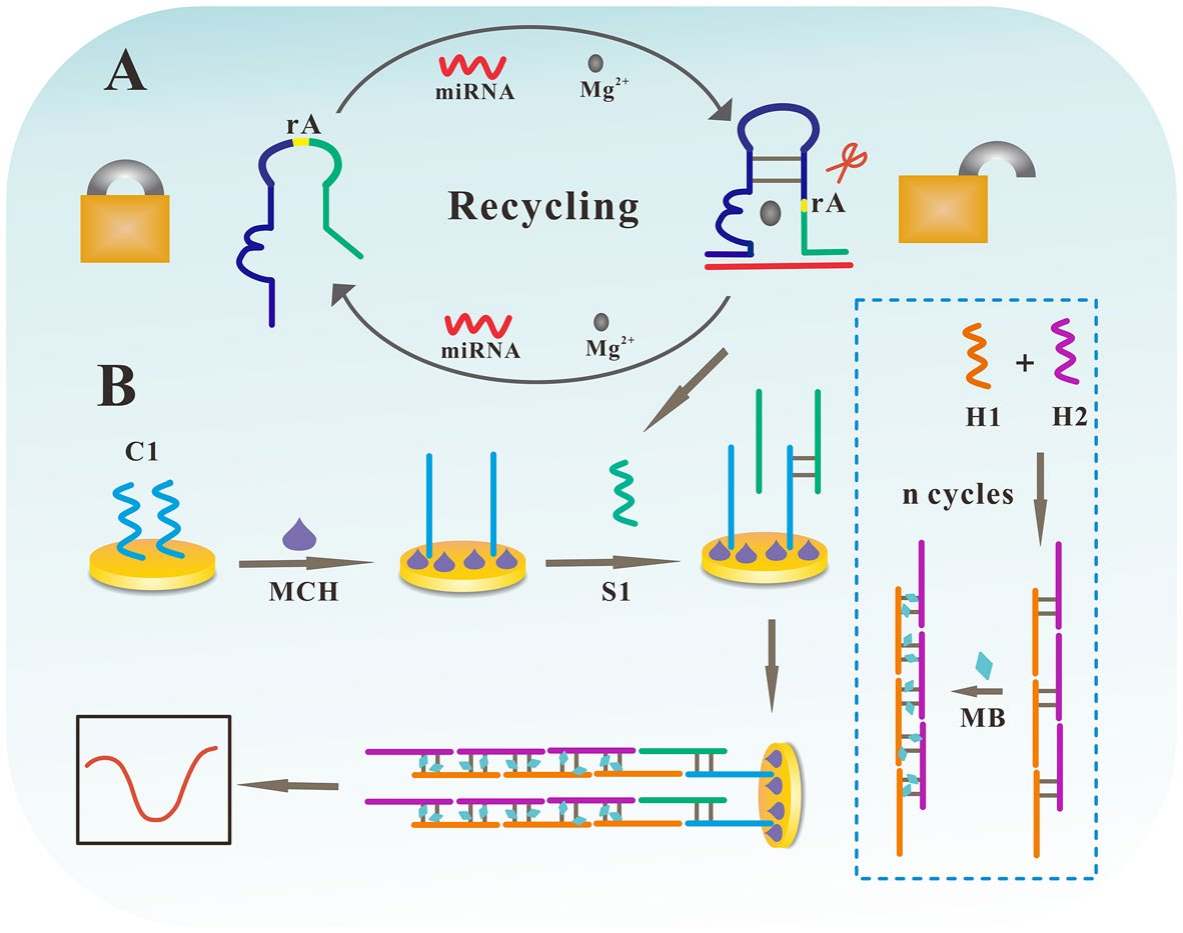
The principle of the electrochemical biosensor for the detection of extracellular vesicles miRNAs. (**A**) The target miRNA keeps in recycling to bind to NAL to release more strands. (**B**) Strand recognition and signal amplification on the electrode for electrochemical measurements.

The gold electrode (GE) was first polished on a microcloth embedded with 0.3 μM alumina powder (Al_2_O_3_), and then ultrasonically cleaned in ethanol and Milli-Q water for 5 min respectively, and then dried at room temperature. Subsequently, the pretreated GE was used to fabricate the electrochemical biosensor as follows (Fig. 1). Firstly, 3 µl of 2 µM capture probe (C1) was dropped on the surface of GE in a constant temperature and humidity chamber at 37 °C for 2 h; Secondly, 3 µl of 1 mM mercaptohexanol (MCH) solution was dropped on the surface of GE at 37 °C for 30 min to eliminate nonspecific adsorption of DNA. After each step, the modified GE was washed with TRIS–HCl (10 mM) solution^29^. Thirdly, 5 µl of different concentrations of target miRNA (0 fM, 1 fM, 10 fM, 10^2^ fM, 10^3^ fM, 10^4^ fM) and 5 µl of Mg^2+^ (20 mM) solution was dipped in 10 µl of NAL (2 µM) solution, respectively, and incubated at 37 °C for 2 h; Next, the mixture dropwise was added to the above-modified electrode and incubated at 37 °C for 1.5 h; And then, 100 µl H1 (2 µM) and 100 µl H2 (2 µM) was hybridized and incubated at 37 °C for 3 h to form DNA tandems. Subsequently, 20 µl of MB (2 mM) solution was added and mixed by shaking at 37 °C for 1 h; Finally, 5 µl of the above solution was added to the electrode and incubated at 37 °C for 1 h; The response signal of MB was recorded in TRIS–HCl solution (10 mM, pH = 7.4) by differential pulse voltammetry (DPV). The above three steps were performed simultaneously and the whole experiment took almost six hours. Reagents cost within one dollar per experiment.

### Electrochemical detection

A three-electrode system consisting of a working electrode (GE with a diameter of 3 mm), a counter electrode (platinum wire) and a reference electrode (Ag/AgCl) was used on a CHI660E electrochemical workstation (CH Instrument, Shanghai, China) for electrochemical detection. The electrochemical properties of the biosensor were analyzed by DPV in a glass vial containing Tris–HCl solution (10 mM, pH = 7.4). DPV was performed in the potential range of − 0.6 V to 0 V, amplitude of 0.05 V, and sensitivity of 1e−5 A/V. We selected the curves with a peak potential of − 0.22 V. All measurements were performed at room temperature.

### Statistical analysis

In this study, we evaluated the established method in terms of sensitivity, specificity, accuracy, and repeatability. All statistical analysis was performed using GraphPad Prism 8.0 (GraphPad Software, San Diego, California USA, www.graphpad.com). Differential expression of miRNA between two independent groups was examined using Mann–Whitney U test or Pearson correlation, with a significance threshold set at a *p* value < 0.05.

### Ethics approval and consent to participate

This study was sought and approved by the Ethics Committee of Fujian Provincial Hospital (Ethics Approval Number: K2018-12-040). Informed consent was obtained from all subjects and/or their legal guardian(s). All methods were carried out in accordance with relevant guidelines and regulations.

## Results

### Feasibility

**Figure 2:**
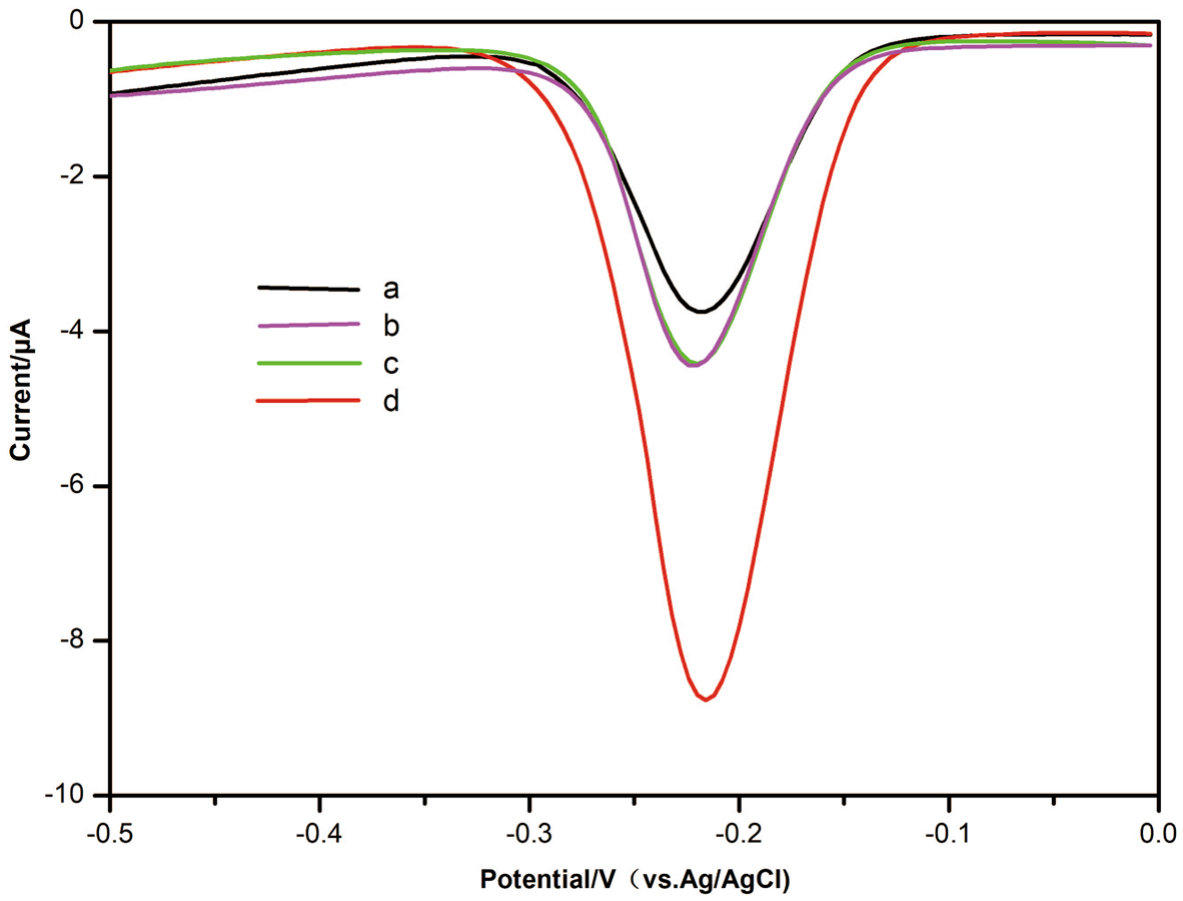
Feasibility characterization of the electrochemical biosensor. The DPV curves of different assembly electrodes in Tris–HCl solution. (**a**) C1/MCH/NAL/DNAzyme/miR-4732-5p/H1/H2/GE; (**b**) C1/MCH/NAL/DNAzyme/ miR-4732-5p/GE; (**c**) C1/MCH/NAL/miR-4732-5p/H1/H2/GE; (**d**) C1/MCH/NAL/DNAzyme/miR-4732-5p/H1/H2/GE. MiR-4732-5p concentrations (from **a** to **d**): 0 fM, 10 fM, 10 fM, 10 fM.

To validate the electrochemical measurements, DPV was used to characterize different assembly processes on Au electrodes. As DPV results shown in Fig. 2, in the absence of miR-4732-5p, NAL and HCR failed to be initiated, and the reduction peak of the blank current signal (curve a) was low. In the presence of the same concentration of miR-4732-5p, the current intensity under the reduction peak adopting only DNAzyme (curve b) or adopting only HCR (curve c) was the same as the blank current signal (curve a) and the current intensity adopting both DNAzyme and HCR (curve d) was significantly higher than those of adopting only DNAzyme (curve b) or adopting only HCR (curve c) (Fig. 2). The DPV results revealed that it was feasible to use the electrochemical biosensor to detect miR-4732-5p.

### Linear experiments

**Figure 3:**
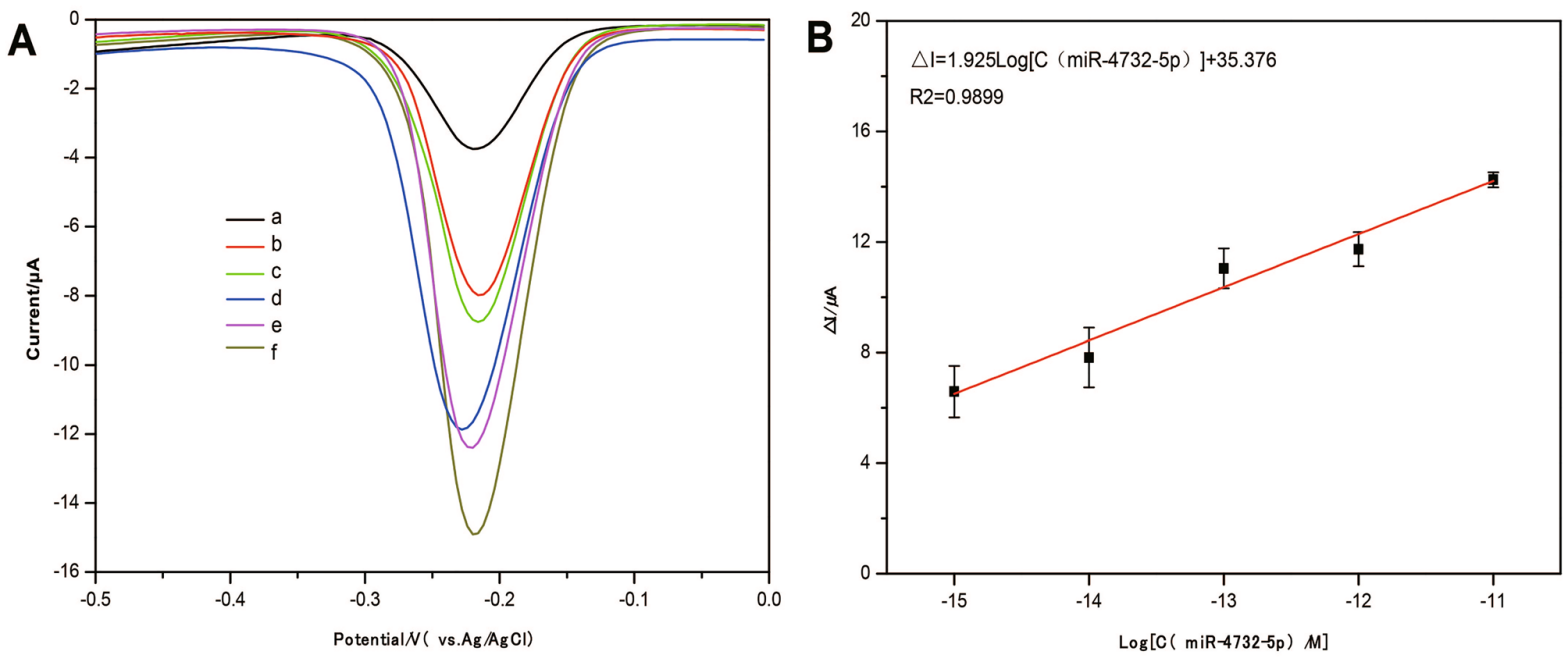
The sensitivity analysis of developed electrochemical biosensor. (**A**) The current signals of different concentrations of miR-4732-5p on the biosensor. miR-4732-5p concentrations (from **a** to **f**): 0 fM, 1 fM, 10 fM, 10^2^ fM, 10^3^ fM, 10^4^ fM. (**B**) The linear curve fitted by the peak current change (ΔI) before and after the addition of different concentrations of miR-4732-5p and the log C_miR-4732-5p_. Error bars represent the standard deviations for measurements taken from three independent experiments.

The dynamic range of this electrochemical biosensor was initially assessed. As shown in Fig. 3A, the current signal amplified as the concentration of miR-4732-5p rose from 0 to 10^4^ fM. The variations in peak current (ΔI, ΔI = I_Sample_ − I_Blank_) before and after adding different concentrations of miR-4732-5p (C_miR-4732-5p_) were recorded and evaluated. Consequently, it was determined in this study to use the range of 1 to 10^4^ fM as the linear curve for the developed electrochemical method to quantify miR-4732-5p levels. The linear curve showed a good linear relationship between the ΔI and the log C_miR-4732-5p_ as illustrated in Fig. 3B, following Eq. (1).

1 $$\Delta {\text{I}} = 1.925\log {\text{ C}}_{{\text{miR - 4732 - 5p}}} + 35.376$$

### Sensitivity

The determination was repeated three times under identical circumstances for the background signal (with an equal volume of ultrapure water), and the limit of detection (LOD) was calculated to be 2.6 × 10^−17^ M by substituting ${\overline{\text{X}}}$ + 3σ (${\overline{\text{X}}}$ is the average value of the background signal and σ is the standard deviation of the background signal) in the linear regression equation as ΔI for the calculation of C_miR-4732-5p_ (C_miR-4732-5p_ was the LOD) (Table 4)^30^. The low detection limit indicated that the method has high sensitivity^33,34^.

**Table 4:** Detection limit of electrochemical biosensor.

	Peak current I _(0 fM)_ (μA)	Peak current I _空白_ (μA)	
Measured value 1	3.404	0.1117	
Measured value 2	3.474		
Measured value 3	3.795		
			*X* = 3.446
			*σ* = 0.170

### Specificity

**Figure 4:**
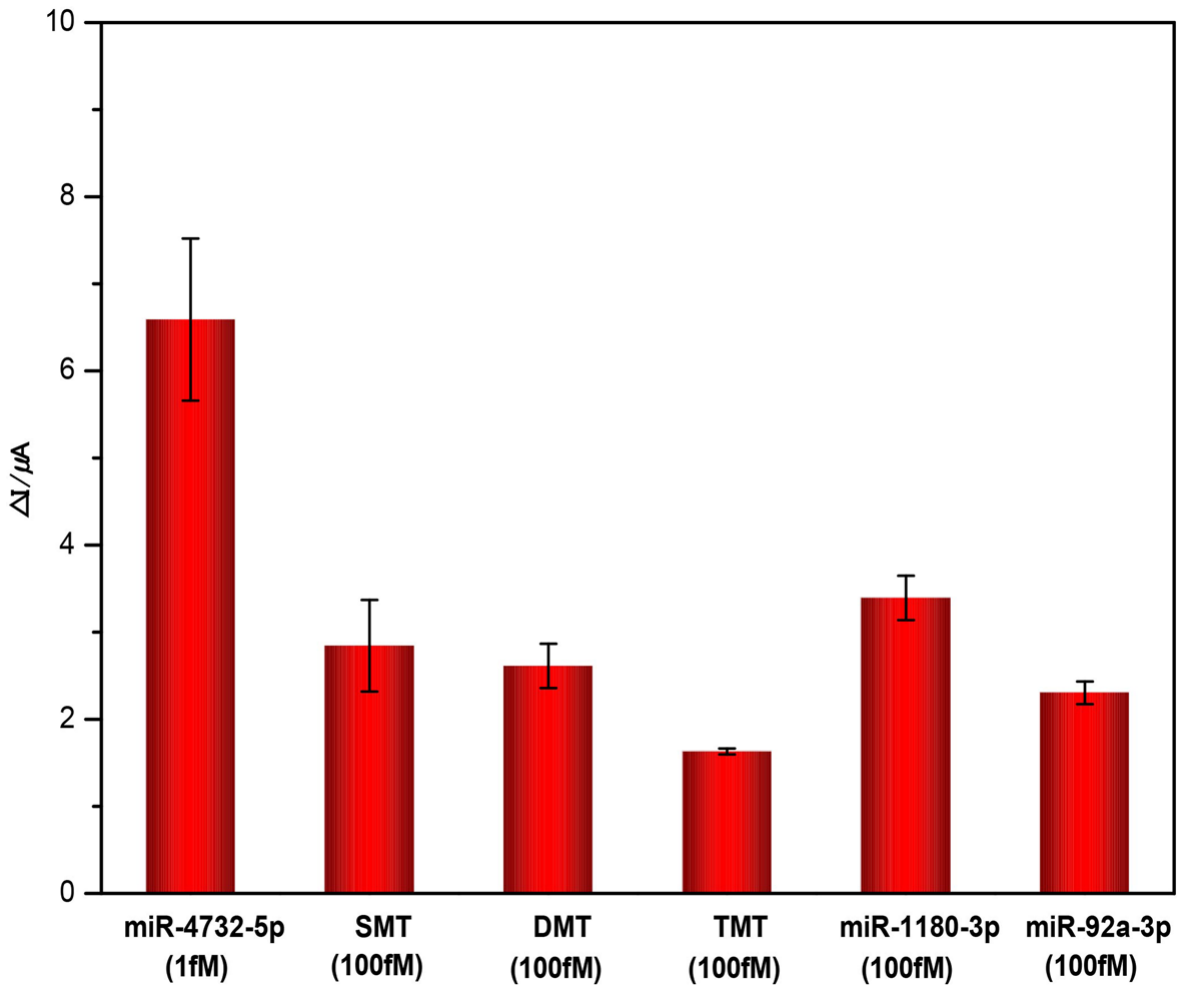
The specificity analysis of the developed electrochemical biosensor towards miR-4732-5p, mismatched miR-4732-5p (SMT, single base mismatched target; DMT, double bases mismatched target; TMT, three bases mismatched target), miR-92a-3p and miR-1180-3p. Error bars represent standard deviations for measurements taken from three independent experiments.

To determine the specificity of the electrochemical biosensor, we detected miR-4732-5p (1 fM), three mismatched miR-4732-5p (100 fM) as well as two extraneous miRNAs of miR-92a-3p (100 fM) and miR-1180-3p (100 fM) using the dual amplification assay. As shown in Fig. 4, the miR-4732-5p (1 fM) produced a peak current change (ΔI) at least 2 times greater than both the mismatched miR-4732-5p (100 fM) and the other two extraneous miRNAs (100 fM).

### Accuracy

In the evaluation of quantitative analysis, the accuracy is usually expressed by the recovery rate. An early LUAD patient specimen was selected as the base sample, 20 μl of base sample and 20 μl of ultrapure water were taken as control samples; 20 μl of base sample and 20 μl of calibrator with a concentration of 200 fM were taken as recovery samples 1; 20 μl of base sample and 20 μl of calibrator with a concentration of 300 fM were taken as recovery samples 2; 20 μl of base sample and 20 μl of calibrator with a concentration of 600 fM were taken as the recovery sample 3; 5 μl of the above control sample and the three recovery samples were taken for detection respectively. Equations (2), (3), and (4) are the calculation formulas of the recovery rate. The results of the recovery experiments, calculated according to the relevant formula, showed that the average recovery of the assay was 97.72% (Table 5), which was between 85 and 115%.

2 $${\text{Recovery}}\;{\text{rate}} = {\text{C}}_{{\text{r}}} /{\text{C}}_{{\text{a}}} \times 100\%$$

3 $${\text{C}}_{{\text{r}}} = {\text{C}}_{{\text{f}}} - {\text{C}}_{{\text{b}}}$$

4 $${\text{C}}_{{\text{a}}} = {\text{C}}_{{\text{s}}} \times {\text{V}}_{{\text{s}}} /({\text{V}}_{{\text{s}}} + {\text{V}}_{{\text{b}}} )$$

**Table 5:** Determination of recovery rate by electrochemical biosensor.

	Recycled samples 1	Recycled samples 2	Recycled samples 3	Basic sample
Initial concentration (fM)	224.39	224.39	456.04	116.41
Recovery concentration (fM)	107.98	107.98	339.63	
Additive concentration (fM)	100	150	300	
Rate of recovery (%)	107.98	71.97	113.21	
Average recovery rate (%)	97.72			

In the formula (2), C_r_ is the recovery concentration and C_a_ is the added concentration. In the formula (3), C_f_ is the final assay concentration and C_b_ is the base sample concentration. In the formula (4), C_s_ is the standard concentration, V_s_ is the standard volume and V_b_ is the base sample volume.

### Accuracy (comparison with RT-qPCR method)

Our pilot study has demonstrated that serum fucosylated extracellular vesicles miR-4732-5p in early LUAD patients is markedly elevated compared to that in BPN patients by RT-qPCR^16^. Herein, we employed the constructed electrochemical biosensor to detect the miR-4732-5p from serum fucosylated extracellular vesicles of early LUAD patients and BPN patients, to evaluate the practical application of electrochemical biosensor as compared with RT-qPCR.

**Figure 5:**
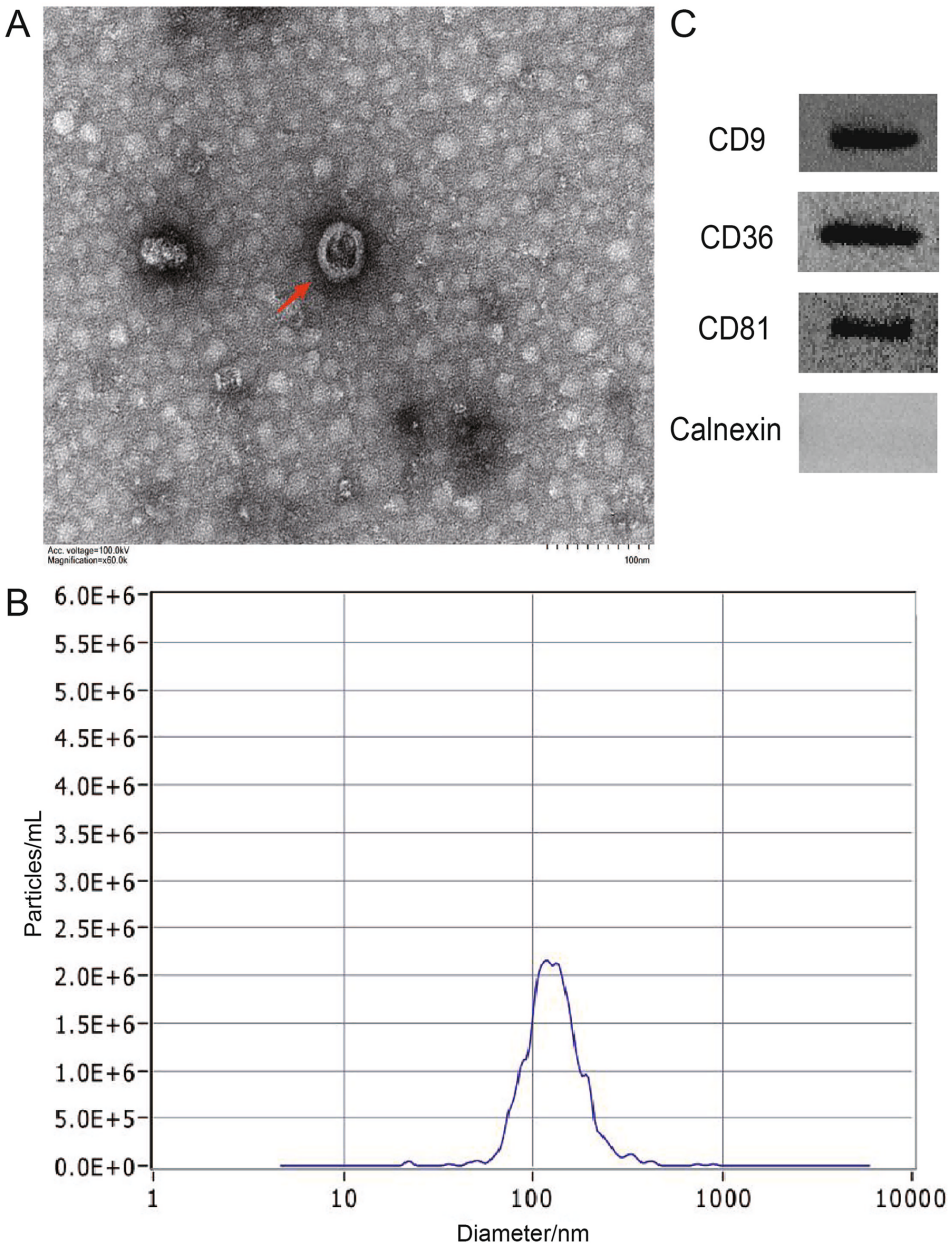
Identification of extracellular vesicles. (**A**) The TEM imaging of extracellular vesicles. (**B**) Particle size distribution of extracellular vesicles. (**C**) WB method for the identification of extracellular vesicles.

A LUAD patient cohort (n = 36; that is 18 for BPN patients and 18 for early LUAD patients) were collected. Firstly, we performed a fucose-captured strategy based on LCA-magnetic beads^16^ to isolate tumor-derived extracellular vesicles in serum, based on aberrantly high fucosylation of membrane surface proteins of extracellular vesicles derived from tumor cells^31^. Subsequently, through TEM, NTA, and Western blotting, fucosylated vesicles isolated from serum were demonstrated to present the extracellular vesicles characteristics, manifested as a typical membrane structure with a size of approximately 30–150 nm, average particle size of 146.0 ± 8.78 nm, positive expressions of CD9, CD63, and CD81 proteins enriched in extracellular vesicles as well as absent expression of calnexin protein as a contamination marker from the endoplasmic reticulum, respectively (Fig. 5A–C).

**Figure 6:**
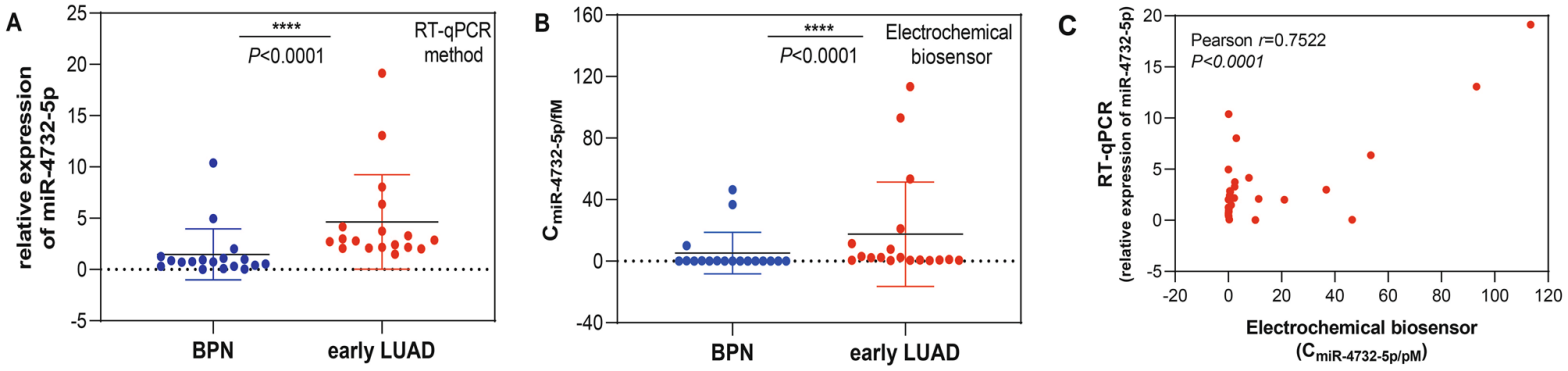
Comparison of electrochemical biosensor and RT-qPCR method. (**A**) The detection of extracellular vesicles miR-4732-5p in BPN and early LUAD patients using the RT-qPCR method. ****p < 0.0001 (Mann–Whitney U test). (**B**) The detection of extracellular vesicles miR-4732-5p in BPN and early LUAD patients using the electrochemical biosensor. ****p < 0.0001 (Mann–Whitney U test). (**C**) The detection of extracellular vesicles miR-4732-5p in BPN and early LUAD patients using the RT-qPCR method and the electrochemical biosensor. *p* < 0.0001 (Pearson correlation).

And then, miR-4732-5p from serum fucosylated extracellular vesicles were detected by electrochemical biosensor and RT-qPCR, respectively. Encouragingly, it was shown that serum fucosylated extracellular vesicles miR-4732-5p levels in early LUAD patients were significantly higher than that in BPN group (*p* < 0.0001) (Fig. 6A,B). Pearson correlation of the detection of extracellular vesicles miR-4732-5p in BPN and early LUAD patients using the RT-qPCR method and the electrochemical biosensor (*p* < 0.0001) (Fig. 6C).

### Reproducibility

Reproducibility refers to the maximum difference between two independent test results at 95% probability level for multiple single test results of the same sample made by the same operator in the same laboratory with the same instrument. In this study, 100 fM and 0 fM miR-4732-5p were repeatedly detected under identical conditions for 5 times, respectively, to assess the reproducibility of the electrochemical biosensor, and the relative standard deviation (RSD) were 9.88% and 9.12% (Table 6). The acceptable range is less than 10%.

**Table 6:** Reproducibility evaluation of electrochemical biosensor.

Sample concentration	S (μA)	X (μA)	RSD (%)
100 fM	1.080	10.934	9.88
0 fM	0.402	4.407	9.12

### Stability

To monitor the stability of the biosensor, we stored the fabricated biosensors in TRIS–HCl solution (10 mM) at 4 °C for 7 days and tested their responses. Serum samples with low, medium, and high concentrations of miR-4732-5p were detected using 3 fabricated biosensors from the same batch after storage for 0 days and 7 days, respectively. The results on the 7th day were compared with those on the 0th day, respectively. The modified electrode could still be used showing nearly 100% signal intensity. The current change after 7 days was maintained to within 95.77% of the original signal (Table 7).

**Table 7:** Stability evaluation of electrochemical biosensor.

	Peak current I_(0 day)_ (μA)	Peak current I_(7 day)_ (μA)	I_(7 day)_/I_(0 day)_ (%)
GE1	15.83	15.16	95.77
GE2	4.793	4.672	97.48
GE3	3.246	3.128	96.36

## Discussion

We constructed an electrochemical biosensor assay for serum fucosylated extracellular vesicles miR-4732-5p detection based on the Mg^2+^-dependent DNAzyme splitting NAL cycle and HCR signal amplification at an electrochemical workstation in this study. Based on the above conditions, we investigated the performance (e.g., sensitivity, specificity, accuracy, reproducibility, and stability) of the electrochemical biosensor using DPV.

In this study, miR-4732-5p obtained from serum fucosylated extracellular vesicles isolated using a fucose-captured strategy was combined with NAL, leading to the splitting of the Mg^2+^-dependent DNAzyme NAL and releasing auxiliary DNA (S1) strands^32^. These S1 strand were then captured by the immobilized C1 strands on the electrode. However, in the absence of the target miRNA, the “lock” remains unpaired with the “key”, preventing the NAL from opening. Subsequently, abundant quantities of H1 and H2 tandems were generated through HCR^33^, and S1 strands hybridized with the H1 and H2 tandems through base complementary pairing. Finally, MB was bonded with the H1 and H2 tandems through π–π stacking^29^, and a signal current was generated when the electrode detected a potential sufficient to induce a redox change in MB^34,35^. These H1, H2 tandems effectively absorbed abundant quantities of MB, enabling the ultrasensitive detection of miRNAs in serum extracellular vesicles^36^.

In recent years, the NAL cycle finds extensive applications in areas such as genetic testing, molecular diagnostics, and biosensors. It not only detects the presence of targeted DNA or RNA, but also allows for the detection of various molecular interactions, such as protein binding and cell recognition. This approach provides an efficient and sensitive tool for biomedical research and clinical diagnostics. As a key, the target miRNA can open the NAL, and activate the catalytic activity of NAL with the assistance of metal ions^32^. Encouragingly, Guo et al. reported an ultrasensitive electrochemical biosensor utilizing HCR signal amplification, achieving an attomoles level detection limit^37^. Given the low and even trace levels of miRNAs in extracellular vesicles, substantial signal amplification is very essential for their detection^38^. Therefore, our study has similar performance with the related detection methods published before.

To further evaluate the performance of our approach, we conducted an assessment, taking into consideration commonly reported evaluation metrics from the literature, such as sensitivity, specificity, accuracy, reproducibility, and stability. The dynamic range of this electrochemical biosensor was first analyzed. The linear curve showed a good linear relationship between the peak current change (ΔI) and the logarithm of C_miR-4732-5p_ (log C_miR-4732-5p_). Based on the 3σ rule, the LOD was calculated. We noted that the high sensitivity was achieved through the dual amplification reactions of the S1 strands and the H1 and H2 tandems. The sensitivity of our study is higher than the similar assays published before^35^. To determine the specificity of the electrochemical biosensor, we challenged the amplification assay with mismatched miR-4732-5p and different extraneous miRNAs. The electrochemical biosensor showed high specificity for miR-4732-5p and was consistent with the similar assays published before^36^, as miR-4732-5p was critical for generating S1 strands that were complementarily paired with H1 and H2 tandems. In the evaluation of quantitative analysis, the results of the recovery experiments indicated the good accuracy of the method for the detection of miR-4732-5p. Additionally, the detection of miR-4732-5p was repeated under identical conditions to assess the reproducibility of the biosensor. It was shown that the developed electrochemical biosensor had good reproducibility. In the evaluation of stability, it was also shown that the developed electrochemical biosensor was also highly stable and consistent with the similar assays published before^36^. To evaluate practical clinical applications, we employed the proposed electrochemical biosensor to detect the miR-4732-5p from serum fucosylated extracellular vesicles of early LUAD patients and BPN patients. It also validated the potential practical value of using this biosensor for miR-4732-5p analysis, unearthing the perspective of constructed electrochemical biosensor as an alternative approach of RT-qPCR for miR-4732-5p detection.

Although our approach has been evaluated against common performance metrics as reported in the literature, there are still some limitations that need to be addressed for further improvement. During the evaluation process, certain constraints, or challenges such as sample handling, specificity, and signal amplification may arise. To enhance the reliability and efficacy of our method, we have devised plans to tackle these issues in our upcoming research. Regarding sample handling, we aim to explore more refined or automated solutions to enhance experimental reproducibility and accuracy. For specificity concerns, we will optimize and refine our approach to address potential cross-reactivity or non-specific hybridization. Furthermore, we will further enhance our signal amplification strategies to improve detection sensitivity and accuracy. Additionally, we plan to expand the scale of our sample dataset by including miRNA detection in glycosylated vesicles and early diagnosis of other related diseases. By testing and validating with a larger set of samples, we aspire to further validate the feasibility and robustness of our method. In conclusion, we are aware of the existing room for improvement in our current approach and plan to enhance its performance and application prospects by addressing these challenges and broadening the scope of experimental validation.

## Conclusions

To the best of our knowledge, we firstly construct a label-free, enzyme-free and ultrasensitive electrochemical biosensor platform for serum fucosylated extracellular vesicles miR-4732-5p detection in this study with the priorities of simple operation, low cost, portability. It is expectable that this approach based on electrochemical biosensor platform might be applied for the efficient and reliable detection of more serum extracellular vesicles miRNAs related to early LUAD, contribute to the improvement of the efficiency of serological screening for early LUAD, thereby reducing patient mortality.

### Supplementary Information

Supplementary Information 1.

Supplementary Table 1.

Supplementary Table 2.

## Data Availability

Data is provided within the manuscript or supplementary information files.
