# Supplementary figures and images for: Detection of fucosylated extracellular vesicles miR-4732-5p related to diagnosis of early lung adenocarcinoma by the electrochemical biosensor
Source: Sci Rep. 2024 May 16;14:11217. doi: 10.1038/s41598-024-61060-z (PMC11099009; doi:10.1038/s41598-024-61060-z)

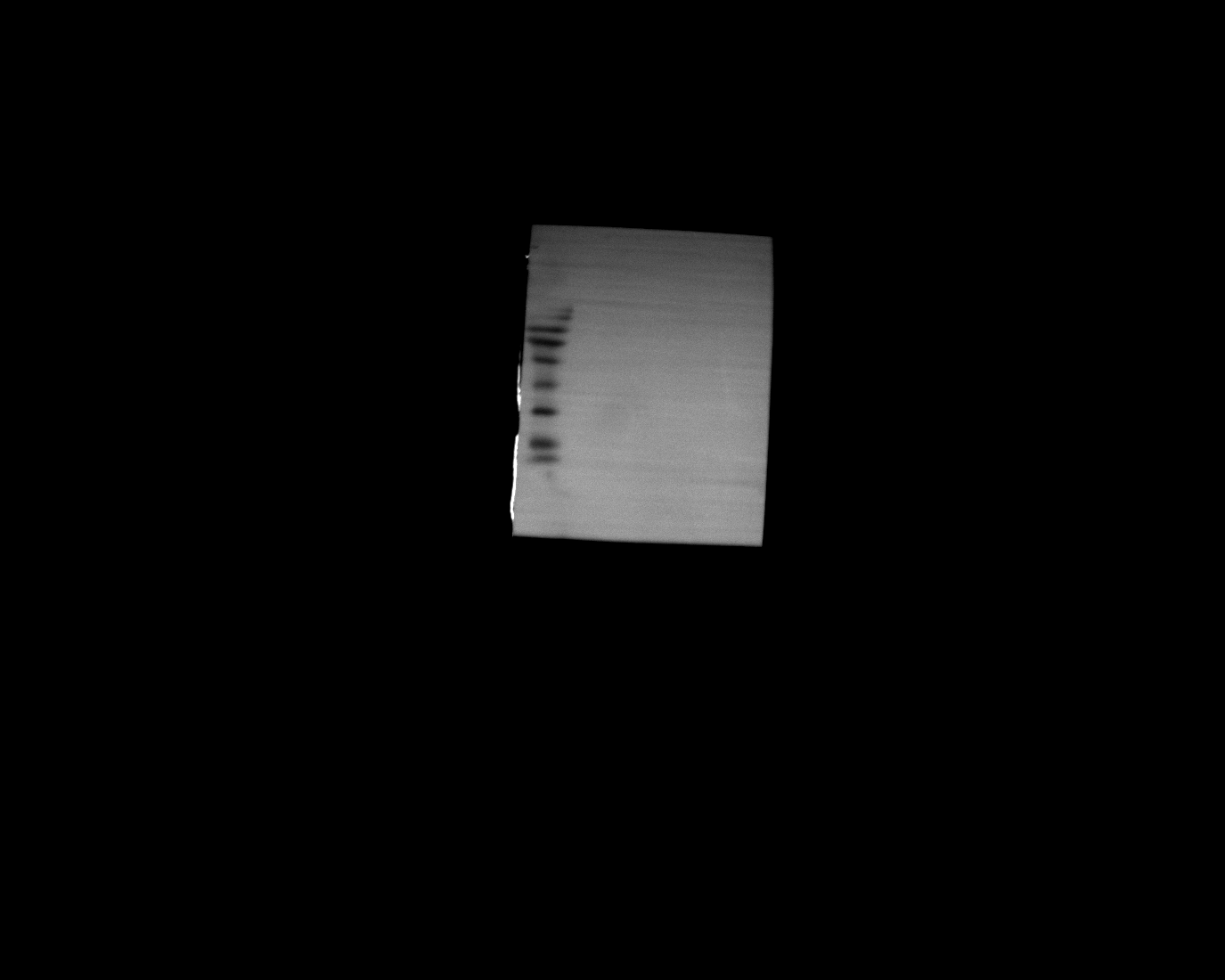

Supplement: Supplementary file 1 — Supplementary Information 1. [file 41598_2024_61060_MOESM1_ESM.zip › Supplementary Material/wb/Calnexin__.tif]

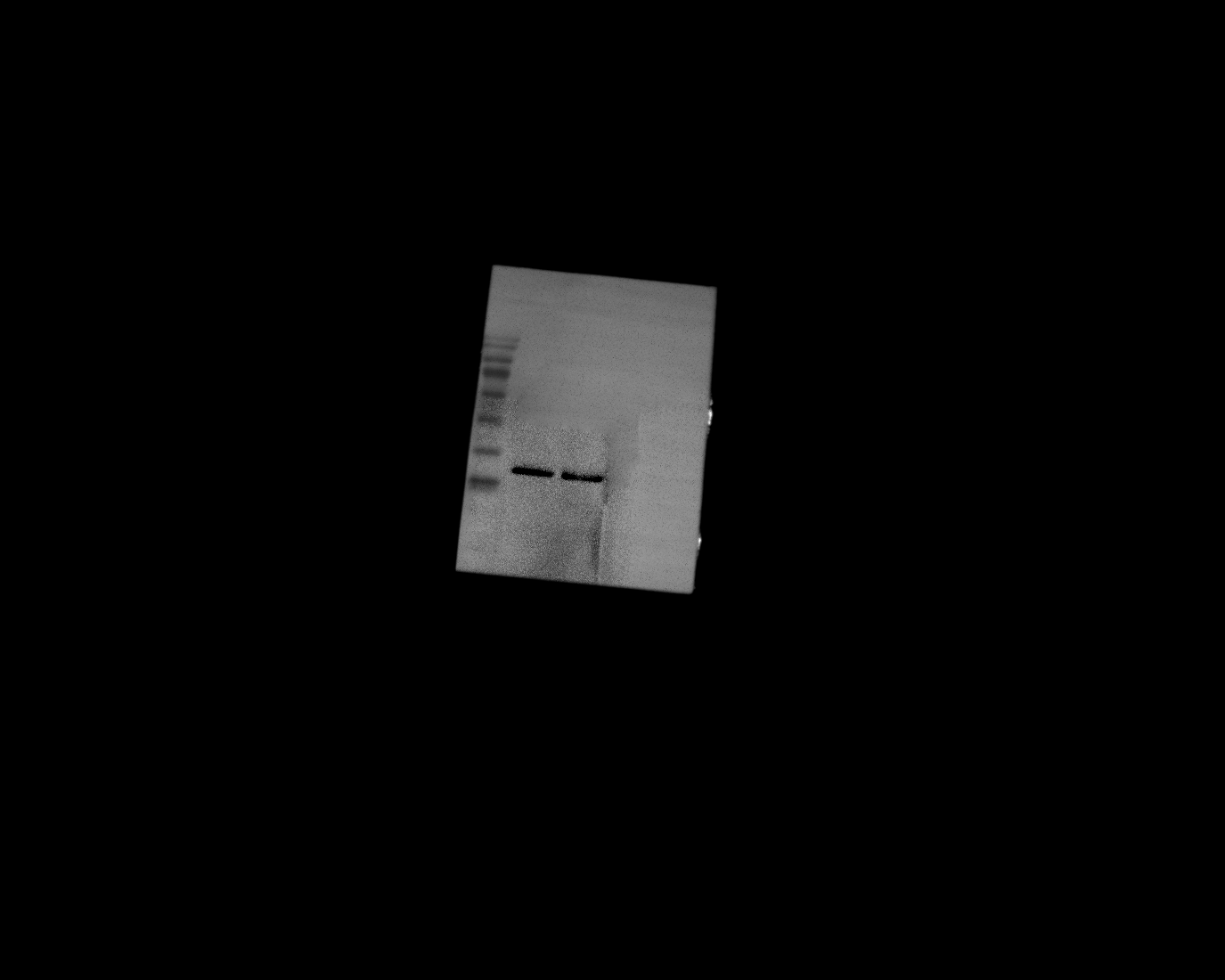

Supplement: Supplementary file 1 — Supplementary Information 1. [file 41598_2024_61060_MOESM1_ESM.zip › Supplementary Material/wb/cd63.tif]

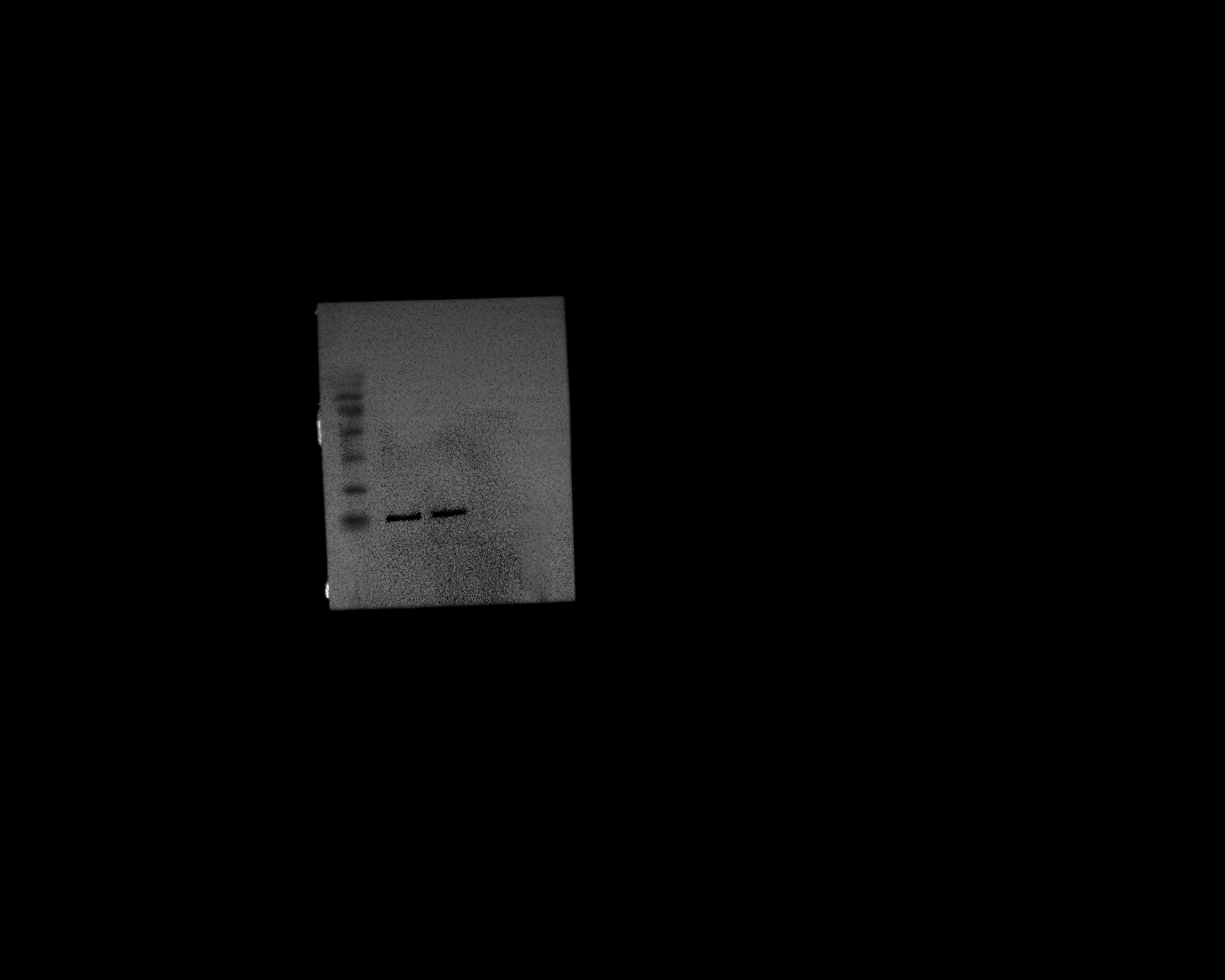

Supplement: Supplementary file 1 — Supplementary Information 1. [file 41598_2024_61060_MOESM1_ESM.zip › Supplementary Material/wb/cd81.tif]

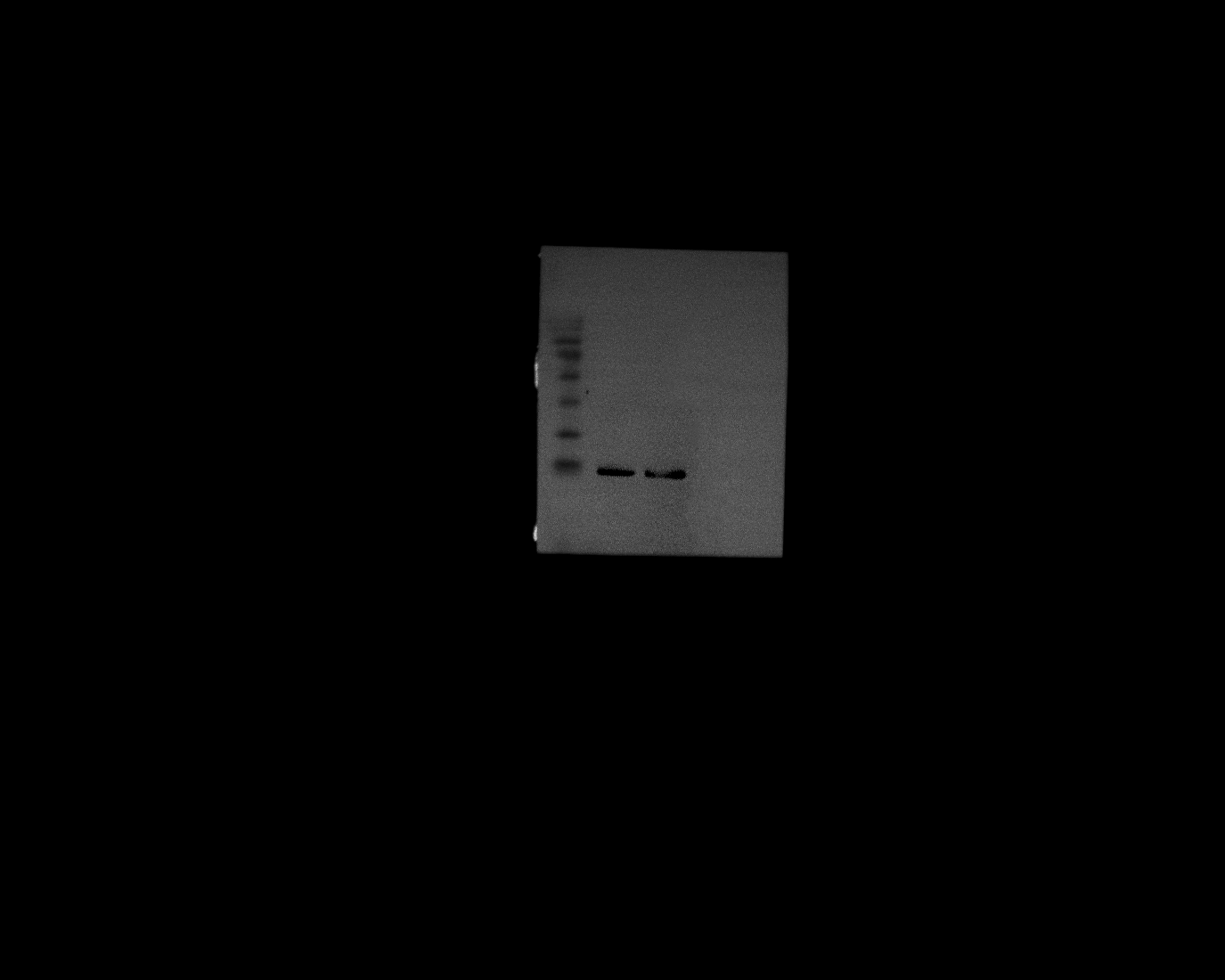

Supplement: Supplementary file 1 — Supplementary Information 1. [file 41598_2024_61060_MOESM1_ESM.zip › Supplementary Material/wb/cd9.tif]
