# Supplementary material for: Detection of fucosylated extracellular vesicles miR-4732-5p related to diagnosis of early lung adenocarcinoma by the electrochemical biosensor
Source: Sci Rep. 2024 May 16;14:11217. doi: 10.1038/s41598-024-61060-z (PMC11099009; doi:10.1038/s41598-024-61060-z)
Supplement: Supplementary file 2 — Supplementary Table 1. [file 41598_2024_61060_MOESM2_ESM.docx]

Table S1. The program for isolating Fucosylated extracellular vesicles and the schematic of the extracellular-vesicle extraction procedure

| Step | Well | Solution | Volume  (µL) | Mixing speed | Mixing time  (minutes) | Precipitation time  (seconds) |
| --- | --- | --- | --- | --- | --- | --- |
| MIX | 1 | MBL | 750 | low | 1 | 240 |
| WASH | 2 | WBL | 600 | low | 1 | 1 |
| ELUTE | 3 | EBL | 250 | low | 1 | 240 |

|  | 1 | 2 | 3 | 4 | 5 | 6 | 7 | 8 | 9 | 10 | 11 | 12 |
| --- | --- | --- | --- | --- | --- | --- | --- | --- | --- | --- | --- | --- |
| A |  |  |  |  |  |  |  |  |  |  |  |  |
| B |  |  |  |  |  |  |  |  |  |  |  |  |
| C |  |  |  |  |  |  |  |  |  |  |  |  |
| D |  |  |  |  |  |  |  |  |  |  |  |  |
| E |  |  |  |  |  |  |  |  |  |  |  |  |
| F |  |  |  |  |  |  |  |  |  |  |  |  |
| G |  |  |  |  |  |  |  |  |  |  |  |  |
| H |  |  |  |  |  |  |  |  |  |  |  |  |

MBL WBL EBL MBL WBL EBL

500μL 600μL 250μL 500μL 600μL 250μL

Abbreviations: MBL, LCA coupled magnetic beads solution; WBL, washing solution; EBL, elution buffer.
