# Supplementary material for: Detection of fucosylated extracellular vesicles miR-4732-5p related to diagnosis of early lung adenocarcinoma by the electrochemical biosensor
Source: Sci Rep. 2024 May 16;14:11217. doi: 10.1038/s41598-024-61060-z (PMC11099009; doi:10.1038/s41598-024-61060-z)
Supplement: Supplementary file 3 — Supplementary Table 2. [file 41598_2024_61060_MOESM3_ESM.docx]

Table S2. The program for isolating EVs-derived RNA and the schematic of the procedure

| Step | Well | Solution | Volume  (µL) | Mixing speed | Temp.  ℃ | Mixing time  (minutes) | Precipitation time  (seconds) |
| --- | --- | --- | --- | --- | --- | --- | --- |
| MIX1 | 1 | LB&MB | 800/16 | FAST | 0 | 5 | 1200 |
| MIX2 | 1 | LB&MB | 800/16 | FAST | 0 | 5 | 1200 |
| MIX3 | 1 | LB&MB | 800/16 | STOP | 0 | 0 | 0 |
| WASH1 | 2 | WB1 | 500 | LOW | 0 | 0 | 0 |
| WASH2 | 3 | WB2 | 800 | LOW | 0 | 0 | 0 |
| ELUTE | 6 | EB | 50 | Media | 60 | 5 | 240 |

|  | 1 | 2 | 3 | 4 | 5 | 6 | 7 | 8 | 9 | 10 | 11 | 12 |
| --- | --- | --- | --- | --- | --- | --- | --- | --- | --- | --- | --- | --- |
| A |  |  |  |  |  |  |  |  |  |  |  |  |
| B |  |  |  |  |  |  |  |  |  |  |  |  |
| C |  |  |  |  |  |  |  |  |  |  |  |  |
| D |  |  |  |  |  |  |  |  |  |  |  |  |
| E |  |  |  |  |  |  |  |  |  |  |  |  |
| F |  |  |  |  |  |  |  |  |  |  |  |  |
| G |  |  |  |  |  |  |  |  |  |  |  |  |
| H |  |  |  |  |  |  |  |  |  |  |  |  |

50 uL EB

50 uL EB

800 uL WB2

800 uL WB2

500 uL WB1

500 uL WB1

800 uL LB & 16 uLMB

800 uL LB & 16 uLMB

Abbreviations: MB, magnetic beads solution; LB, lysis solution; WB, washing solution; EB, elution buffer.
